# Characterization of US State COVID-19 Vaccine Incentive Programs

**DOI:** 10.1001/jamanetworkopen.2022.35328

**Published:** 2022-10-07

**Authors:** Caroline M. Hogan, Aaron S. Parzuchowski, Xiru Lyu, Jason Goldstick, Kenneth Resnicow

**Affiliations:** 1 Child Health Evaluation and Research Center, Department of Pediatrics, University of Michigan Medical School, University of Michigan, Ann Arbor; 2 University of Michigan National Clinician Scholars Program, Ann Arbor; 3 Department of Internal Medicine, University of Michigan Medical School, University of Michigan, Ann Arbor; 4 US Department of Veterans Affairs, University of Michigan National Clinician Scholars Program, Ann Arbor; 5 Department of Emergency Medicine, University of Michigan Medical School, University of Michigan, Ann Arbor; 6 Department of Health Behavior and Health Education, School of Public Health, University of Michigan, Ann Arbor; 7 Rogel Cancer Center, University of Michigan, Ann Arbor

## Abstract

This cross-sectional study describes and compares key characteristics of state COVID-19 vaccine incentive programs in the US.

## Introduction

Throughout 2021, US states used incentives to promote COVID-19 vaccination.^1^ Although effectiveness evaluations of state-level incentives exist,^2,3^ a broader characterization of these initiatives has not been reported. We describe key features of state-level COVID-19 vaccine incentive programs, the political characteristics of states offering incentives, and the co-incidence of incentives with other COVID-19 mitigation policies.

## Methods

In this cross-sectional study, state COVID-19 vaccine incentives were defined as any state government–supported prize or payment requiring COVID-19 vaccination for eligibility. Incentives were compiled from the National Governors Association and internet searches between September 25 and December 1, 2021, and the resulting list is available for reader review.^4^

Given substantial incentive heterogeneity, we focused on 3 incentive types: lotteries with cash prizes, guaranteed cash payments (excluding programs that only offered gift cards for specific retailers or purchase categories, like groceries), and scholarships. We included incentives offered to the general public (rather than specific subpopulations, like state employees). We documented each incentive program’s duration, target population (adults or children), and the number and monetary value of prizes or payments. Finally, we documented each state’s gubernatorial or mayoral (Washington, DC) political affiliation and whether the state implemented mask requirements, travel restrictions, and/or vaccine mandates^5,6^ in 2021, using Fisher exact test to determine significant differences (2-sided *P* < .05). Analyses were performed using Excel, version 16.64 (Microsoft Corporation). The study followed STROBE reporting guidelines.

## Results

Twenty-seven states including the District of Columbia (53%) offered at least 1 lottery, guaranteed cash payment, and/or scholarship incentive to the general public, covering 55% of the US population. Twenty-one states (41%) offered 2 or more incentive types. We identified 24 lottery programs in 23 states (45%), 19 guaranteed cash payment programs in 12 states (24%), and 23 scholarship programs in 19 states (37%) (Table).

**Table.** US State Use of COVID-19 Vaccine Incentives^a^

State	Political affiliation^c^	Nonincentive COVID-19 mitigation policy^d^	Incentive type^b^		
			Lottery	Guaranteed cash payment	Scholarship
Alabama	R	Yes	No	No	No
Alaska	R	Yes	Yes	No	Yes
Arizona	R	No	No	No	No
Arkansas	R	Yes	Yes	No	No
California	D	Yes	Yes	Yes	No
Colorado	D	Yes	Yes	No	Yes
Connecticut	D	Yes	No	No	No
Delaware	D	Yes	Yes	Yes	Yes
District of Columbia	D	Yes	No	Yes	Yes
Florida	R	No	No	No	No
Georgia	R	No	No	No	No
Hawaii	D	Yes	Yes	No	No
Idaho	R	No	No	No	No
Illinois	D	Yes	Yes	No	Yes
Indiana	R	Yes	No	No	No
Iowa	R	Yes	No	No	No
Kansas	D	Yes	No	Yes	No
Kentucky	D	Yes	Yes	No	Yes
Louisiana	D	Yes	Yes	Yes	Yes
Maine	D	Yes	Yes	No	No
Maryland	R	Yes	Yes	No	Yes
Massachusetts	R	Yes	Yes	No	Yes
Michigan	D	Yes	Yes	Yes	Yes
Minnesota	D	Yes	No	Yes	Yes
Mississippi	R	Yes	No	No	No
Missouri	R	No	Yes	No	Yes
Montana	R	Yes	No	No	No
Nebraska	R	No	No	No	No
Nevada	D	Yes	Yes	No	Yes
New Hampshire	R	Yes	No	No	No
New Jersey	D	Yes	No	No	No
New Mexico	D	Yes	Yes	Yes	No
New York	D	Yes	Yes	No	Yes
North Carolina	D	Yes	Yes	Yes	Yes
North Dakota	R	Yes	No	No	No
Ohio	R	Yes	Yes	No	Yes
Oklahoma	R	No	No	No	No
Oregon	D	Yes	Yes	No	Yes
Pennsylvania	D	Yes	No	No	No
Rhode Island	D	Yes	Yes	No	No
South Carolina	R	No	No	No	No
South Dakota	R	No	No	No	No
Tennessee	R	No	No	No	No
Texas	R	Yes	No	No	No
Utah	R	Yes	No	No	No
Vermont	R	Yes	No	No	No
Virginia	D	Yes	No	No	No
Washington	D	Yes	Yes	Yes	Yes
West Virginia	R	Yes	Yes	Yes	Yes
Wisconsin	D	Yes	No	Yes	No
Wyoming	R	Yes	No	No	No
Total No. of states	NA	41	23	12	19

Abbreviations: D, Democratic Party; NA, not applicable; R, Republican Party.

^a^ State use of 3 types of COVID-19 vaccine incentives (lotteries with cash prizes, guaranteed cash payments, and scholarships) from January 1 to December 1, 2021.

^b^ Which incentive types, if any, states implemented to promote COVID-19 vaccination.

^c^ The political party affiliation of the state’s governor (or mayor for Washington, DC) in 2021.

^d^ Whether states implemented at least 1 COVID-19 mitigation policy (mask requirements, travel restrictions, and/or vaccine mandates) in 2021.

### Lotteries

There were 24 lottery programs with cash prizes; 16 (67%) had multiple prize tiers. We identified 1248 individual lottery prizes with a median prize value of $10 000 (minimum, $20; maximum, $5 000 000; mean, $76 405). Adults were eligible for prizes in 21 programs (88%) and children in 3 (13%). The median duration of lotteries with known end dates was 40 days (23 [96%] programs).

### Guaranteed Cash Payments

The median individual payment value among 19 cash incentive programs was $100 (minimum, $10; maximum, $200; mean, $77). Cash payments were targeted to adults (17 [89%] programs) and children (15 [79%] programs); 1 program (5%) had unclear age eligibility. The median duration of cash programs with known end dates was 31 days (16 [84%] programs).

### Scholarships

Of the 23 identified scholarship programs, 16 (70%) had documented values encompassing 643 individual prizes. The median scholarship value was $10 000 (minimum, $5000; maximum, $300 000; mean, $29 605). Children were eligible for all programs; 5 (22%) also included adults. The median duration of scholarship programs was 52 days.

### State Politics and Nonincentive COVID-19 Mitigation Policies

Twenty (83%) of 24 Democrat-led states offered at least 1 lottery, guaranteed cash payment, and/or scholarship incentive vs 7 (26%) of 27 Republican-led states (*P* < .001). Twenty-six (96%) of the 27 states with incentives implemented at least 1 nonincentive COVID-19 mitigation policy vs 15 (63%) of the 24 nonincentive states (*P* = .004).

## Discussion

This cross-sectional study describes 3 COVID-19 vaccine incentives (lotteries, guaranteed cash payments, and scholarships) broadly used in the US in 2021. Of note, many states offered a heterogeneous collection of other and/or subgroup incentives, such as professional racetrack test drives, free marijuana, and reduced prison sentences.

Political differences existed in states’ incentive use, and states with incentives were more likely to use other COVID-19 mitigation policies. Ongoing efforts to evaluate vaccine incentive effectiveness may be complicated by incentive heterogeneity and co-occurrence with other state-, county-, city-, employer-, and insurer-level incentives. Study limitations include possible missed incentives and overall underreporting of incentive use due to exclusion of nonlottery, cash payment, and scholarship incentives and incentives offered to subgroups, such as state employees.
